# Seltene Ätiologie der Otitis externa: Manifestation eines Myelosarkoms im Felsenbein

**DOI:** 10.1007/s00106-026-01731-7

**Published:** 2026-02-12

**Authors:** V. Ihle, E. Goldberg-Bockhorn, E. Lemaitre, V. I. Gaidzik, T. K. Hoffmann, A. S. Kuhn

**Affiliations:** 1 Klinik für Hals-Nasen-Ohrenheilkunde, Kopf- und Halschirurgie, Universitätsklinikum Ulm, Frauensteige 12, 89075 Ulm, Deutschland; 2 Institut für Pathologie, Universitätsklinikum Ulm, Albert-Einstein-Allee 23, 89081 Ulm, Deutschland; 3 Klinik für Innere Medizin III, Universitätsklinikum Ulm, Albert-Einstein-Allee 23, 89081 Ulm, Deutschland

**Keywords:** Myelosarkom, Otalgie, Mastoidektomie, Chlorom, Gehörgangsstenose, Sarcoma, myeloid, Otalgia, Mastoidectomy, Chloroma, External auditory canal stenosis

## Abstract

Ein 45-jähriger Patient präsentierte sich mit einer ausgeprägten Gehörgangsschwellung sowie Symptomen einer Otitis externa, die auf konservative Behandlungsversuche nicht ansprachen. Im weiteren Verlauf trat eine begleitende Mastoiditis auf, woraufhin eine Mastoidektomie erfolgte. Die pathologische Untersuchung ergab keinen wegweisenden Befund. Bei ausbleibender Besserung führten zuletzt eine Gehörgangsbiopsie und Mastoidrevision mit erneuter pathologischer Untersuchung zur Diagnose eines Myelosarkoms, im Sinne eines Rezidivs einer zuvor in Remission befindlichen akuten myeloischen Leukämie (AML).

## Abstract (en)

A 45-year-old male patient presented with pronounced swelling of the external auditory canal and symptoms of otitis externa which did not respond to conservative treatment. Due to concomitant mastoiditis, mastoidectomy was performed in the later course, which did not provide conclusive histological results. In the absence of improvement, biopsy of the external auditory canal and mastoid revision with repeated pathological analysis were carried out. Ultimately, a myeloid sarcoma representing a relapse of previously remitted acute myeloid leukemia (AML) was identified.

## Anamnese

Ein 45-jähriger Patient stellte sich wiederholt mit dem Bild einer Otitis externa in der Ambulanz vor. Er berichtete über pulsierende Otalgie, erhöhte Temperatur sowie eine subjektive Hörminderung auf dem linken Ohr. Symptome wie Tinnitus, Otorrhö oder Schwindel wurden verneint. Ein Jahr zuvor war bei dem Patienten eine akute myeloische Leukämie (AML) diagnostiziert worden, die sich nach Induktionschemo- und nachfolgender Konsolidierungstherapie in vollständiger Remission befand. Weitere Vorerkrankungen bestanden nicht.

## Befund

**Abb. 1:**
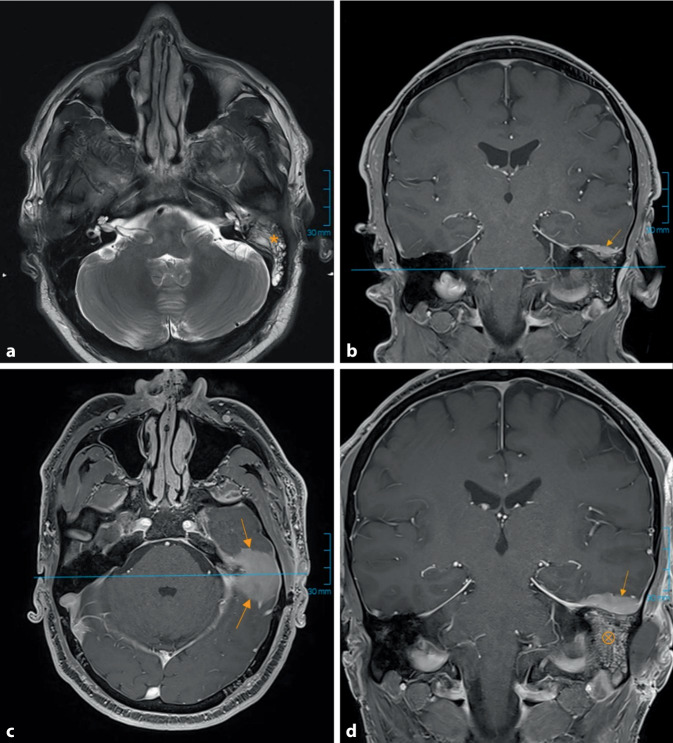
MRT des Schädels vor der ersten Mastoidektomie (**a,** **b**) und vor der Revisionsmastoidektomie (**c,** **d**). In der initialen MRT zeigte sich in der T2-Sequenz (**a**) eine ausgeprägte Flüssigkeitsverlegung der Mastoidzellen mit randlich betonter Kontrastmittelaufnahme sowie Diffusionsstörungen als Zeichen der Mastoiditis (*). Darüber hinaus fiel eine flächige Duraverdickung angrenzend an das linke Felsenbein auf (**b**, T1-VIBE-Sequenz, „volumetric interpolated breath-hold examination“, *Pfeile*). Diese wurde als fokale Meningitis ohne Einschmelzungszeichen gewertet. In der Verlaufsbildgebung ließ sich eine weichteilige, KM-aufnehmende Verlegung der Mastoidhöhle mit zunehmend schlecht abgrenzbarer knöcherner Deckung erkennen (**d**, T1 VIBE, *⊗*). Unmittelbar angrenzend zeigte sich eine progrediente Weichgewebsvermehrung in der mittleren und hinteren Schädelgrube (**c,** **d**, T1 VIBE, *Pfeile*)

Der linke Gehörgang zeigte sich deutlich geschwollen und gerötet bei blandem Befund des Trommelfells. Angefertigte Abstriche erbrachten keinen Nachweis einer bakteriellen oder mykotischen Besiedlung. In der Tonschwellenaudiometrie wurde eine kombinierte Schwerhörigkeit mit einer Schallleitungskomponente von bis zu 40 dB gemessen. Zur Abklärung eines möglichen Diabetes mellitus als Risikofaktor einer Otitis externa maligna wurde der HbA1c-Wert bestimmt, der normwertig ausfiel. Ebenso zeigte sich bei normaler Leukozytenzahl lediglich eine diskrete Erhöhung des C-reaktiven Proteins (CRP). Die durchgeführten Differenzialblutbilder bestätigten weiterhin eine hämatologische Remission der akuten myeloischen Leukämie (AML). Bei mangelndem Ansprechen auf antibiotische Ohrentropfen sowie intravenöse Antibiose mit Piperacillin/Tazobactam wurde im Verlauf eine CT durchgeführt. Diese legte den Verdacht auf eine begleitende Mastoiditis mit intrakraniellem Durchbruch nahe. Eine ergänzende MRT ergab neben einer Duraverdickung am Felsenbein jedoch keinen Anhalt auf einen intrakraniellen Abszess (Abb. 1a, b).

## Therapie und Verlauf

**Abb. 2:**
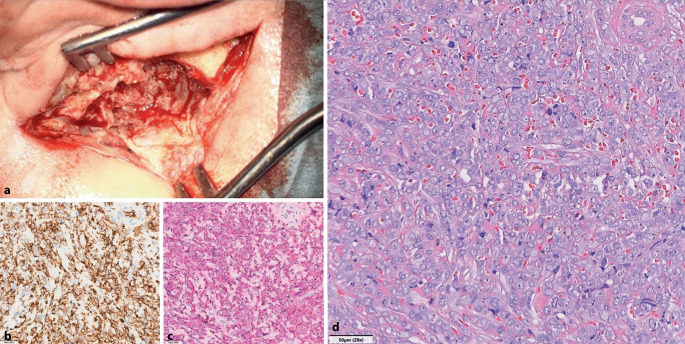
Intraoperativer Situs (**a**) und histologischer Befund (**b–d**) der Revisionsmastoidektomie. Im Rahmen der Mastoidrevision über einen retroaurikulären Zugang präsentierte sich schwartiges, tumoröses und stark blutendes Gewebe, welches die gesamte Mastoidhöhle ausfüllte (**a**). In der Histologie zeigte sich ein Zellinfiltrat mit großen, blastären Zellen, die prominente Nukleolen und ein aufgelockertes, vesikuläres Chromatin aufwiesen (HE-Färbung, Vergr. 20:1; **d**). Die blastären Zellen zeigten eine membranöse Expression von CD34 (CD34-Färbung, Vergr. 20:1; **b**) und eine zytoplasmatische Expression von Myeloperoxidase (MPO; MPO-Färbung, Vergr. 20:1; **c**)

Zur Sanierung des Herdbefunds erfolgte eine Mastoidektomie. Bei überwiegend nekrotischem Gewebe der intraoperativ gewonnenen Proben ergab sich in der pathologischen Beurteilung kein wegweisender Befund. Eine Kontrollbildgebung via MRT zeigte ein beginnendes Ödem des Temporallappens. Unter dem Verdacht einer subakuten Mastoiditis mit meningealer Beteiligung erhielt der Patient eine antibiotische Therapie mit Piperacillin/Tazobactam. Die Entlassung erfolgte mit Empfehlung zur Applikation von antiseptischen Ohrentropfen bei anhaltender Gehörgangsschwellung. Im Rahmen der folgenden Vorstellungstermine zeigte sich die Schwellung progredient. Zur weiteren Diagnostik erfolgte eine Probeexzision aus dem Gehörgang. Noch vor Erhalt des pathologischen Befunds wurde der Patient bei akuter Schmerzexazerbation erneut stationär aufgenommen. Die Laboruntersuchungen sowie peripheren Blutkulturen zeigten keine Auffälligkeiten. Bei magnetresonanztomographischem Anhalt für einen beginnenden Hirnabszess (Abb. 1c, d) erfolgte die intravenöse Antibiose nach interdisziplinärer Absprache leitliniengerecht mit Meropenem/Vancomycin. Des Weiteren wurde eine Revisions-Mastoidektomie zur erneuten Histologiegewinnung durchgeführt. Intraoperativ zeigte sich das Mastoid mit tumorartigem Gewebe durchsetzt, das bereits infiltrierend in Richtung Temporalpol wuchs (Abb. 2a). Die pathologische Aufarbeitung der Gehörgangsbiopsie ergab schließlich den Nachweis eines Myelosarkoms, das als extramedulläres Rezidiv der zuvor in kompletter Remission (CR) befindlichen AML auftrat. Der Patient wurde postoperativ durch die Kollegen der Hämatoonkologie weiterbetreut. Eine durchgeführte Durchflusszytometrie aus dem Knochenmark zeigte einen Blastenanteil von unter 5 % bei ähnlichem Immunphänotyp mit aberranter Expression von sCD19 wie bei initialer Diagnosestellung der AML. Der Patient erhielt eine Salvagetherapie gemäß dem FLAVIDA-Regime, unter der eine CR erzielt wurde. Daraufhin erfolgte eine allogene hämatopoetische Stammzelltransplantation. Unter adäquater onkologischer Therapie heilte der Ohrbefund vollständig aus. Die Schallleitungsschwerhörigkeit zeigte sich deutlich regredient. Beim letzten HNO-Kontrolltermin, 8 Monate nach AML-Rezidiv, präsentierte sich der Patient in einem guten Allgemeinzustand.

## Diskussion

Nach der International Consensus Classification wird die AML, sofern keine AML-definierende genetische Aberration vorliegt, bei ≥ 20 % Blasten im Knochenmark diagnostiziert [2]. Bei extramedullärer Blasteninfiltration kann sich die AML aber auch als Chlorom beziehungsweise Myelosarkom präsentieren [9]. Das Myelosarkom (MS) beschreibt einen soliden extramedullären Tumor aus myeloischen Blasten, der isoliert auftreten oder mit einer Knochenmarkbeteiligung einhergehen kann [7]. Es steht in Zusammenhang mit verschiedenen hämatologischen Neoplasien und kann häufig als Erstmanifestation oder im Rahmen eines AML-Rezidivs auftreten [1]. Das MS manifestiert sich nur in 4–14 % der Fälle im Kopf-Hals-Bereich, während Lymphknoten (8–55 %) und Bindegewebe (14–35 %) deutlich häufiger betroffen sind [8]. Im Kopf-Hals-Bereich wurden Tumoren in unterschiedlichen Regionen und Geweben beschrieben, wie beispielsweise in der Mundhöhle [1], Nasennebenhöhle [5] und im Os temporale [6]. Typische Symptome bei MS des Schläfenbeins sind, wie auch in der vorliegenden Kasuistik, Otalgie und Hörstörung. Zudem kann auch eine Fazialisparese auftreten [6]. Aufgrund der klinischen Heterogenität und Seltenheit von MS ist die Evidenz für Diagnostik und Therapie bisher begrenzt. Die Diagnose stützt sich primär auf den pathologischen Befund. Histologisch sieht man ein Infiltrat unreifer Zellen mit Störung der Gewebsarchitektur. Immunhistochemisch lässt sich eine Expression von Markern wie CD45, CD33 und Myeloperoxidase (MPO) nachweisen [1, 8]. Ebenso zeigen viele MS eine aberrante Antigenexpression wie beispielsweise des B‑Zell-Markers CD19 oder der T‑Zell-Marker CD4 oder CD7 [4]. Dies führt gemeinsam mit der variablen klinischen Präsentation dazu, dass in bis zu 47 % der Fälle eine Fehldiagnose gestellt wird [3]. Ein bedeutsames Risiko einer verzögerten Diagnosestellung besteht insbesondere in einer beginnenden Knochenmarkbeteiligung, die bei unbehandelten, isolierten MS typischerweise nach ca. 5–12 Monaten auftritt [7]. Die Therapie des MS orientiert sich mangels spezifischer Leitlinien in der Regel an den etablierten AML-Therapieschemata [4]. In einer retrospektiven Kohorte von 17 Patienten mit MS der Kopf-Hals-Region, die über eine Periode von 24 Jahren beobachtet wurden, waren zum letzten Follow-up-Termin 59 % der Patienten an ihrer Erkrankung gestorben [1].

## Fazit für die Praxis

- Das Myelosarkom stellt eine seltene und diagnostisch herausfordernde Manifestation myeloischer Neoplasien wie der AML dar, die sich unter anderem als therapierefraktäre Otitis externa mit Gehörgangsschwellung präsentieren kann.
- Insbesondere bei Patienten mit malignen hämatologischen Erkrankungen in der Vorgeschichte sollte die Indikation zur Biopsie bei fehlendem Therapieansprechen großzügig gestellt werden, um potenziell prognosekritische Erkrankungen frühzeitig erkennen und behandeln zu können.
